# Women’s empowerment and gender equality in agricultural value chains: evidence from four countries in Asia and Africa

**DOI:** 10.1007/s12571-021-01193-5

**Published:** 2021-09-03

**Authors:** Agnes Quisumbing, Jessica Heckert, Simone Faas, Gayathri Ramani, Kalyani Raghunathan, Hazel Malapit, The pro-WEAI for Market Inclusion Study Team, Hazel Malapit, Jessica Heckert, Sarah Eissler, Simone Faas, Elena Martinez, Emily Myers, Audrey Pereira, Agnes Quisumbing, Catherine Ragasa, Kalyani Raghunathan, Deborah Rubin, Greg Seymour

**Affiliations:** 1 International Food Policy Research Institute, Washington, DC USA; 2 International Food Policy Research Institute, New Delhi, India

**Keywords:** Gender, Women’s empowerment, Market inclusion, Value chains, Food systems

## Abstract

Women play important roles at different nodes of both agricultural and off-farm value chains, but in many countries their contributions are either underestimated or limited by prevailing societal norms or gender-specific barriers. We use primary data collected in Asia (Bangladesh, Philippines) and Africa (Benin, Malawi) to examine the relationships between women’s empowerment, gender equality, and participation in a variety of local agricultural value chains that comprise the food system. We find that the value chain and the specific node of engagement matter, as do other individual and household characteristics, but in different ways depending on country context. Entrepreneurship—often engaged in by wealthier households with greater ability to take risks—is not necessarily empowering for women; nor is household wealth, as proxied by their asset ownership. Increased involvement in the market is not necessarily correlated with greater gender equality. Education is positively correlated with higher empowerment of both men and women, but the strength of this association varies. Training and extension services are generally positively associated with empowerment but could also exacerbate the inequality in empowerment between men and women in the same household. All in all, culture and context determine whether participation in value chains—and which node of the value chain—is empowering. In designing food systems interventions, care should be taken to consider the social and cultural contexts in which these food systems operate, so that interventions do not exacerbate existing gender inequalities.

## Introduction

Food systems are the sum of individuals, institutions, and their interactions—from input supply and production of crops, livestock, fish, and other agricultural commodities, to transportation, processing, retailing, wholesaling and preparation of foods, to their consumption and disposal (Fan & Swinnen, 2020). Broader economic processes shape food systems; for example, as economies transform and a higher proportion of GDP is produced by the manufacturing and services sectors, employment shifts from agriculture to non-agriculture, often accompanied by urbanization, migration, and the nutrition transition. Global experience shows that as countries develop, off-farm components of food systems become more important, creating new job opportunities in sectors like food processing and trading (Mueller et al., 2020).

Both men and women participate in food systems, but the nature and extent of their participation varies depending on the structure of the economy and gender norms. Women are actively involved in a range of roles from production and processing to retailing and consumption; they grow and manage crops, tend livestock, work in agribusinesses and food retailing, prepare food for their families, and much more (Malapit et al., 2020a). But women’s contributions to food systems are often not formally recognized or appropriately valued. Women frequently face constraints that prevent them from engaging on equitable terms; in many countries, women have less schooling than men, control fewer resources, have less decision-making power over household income, and face time constraints because of their triple burden of productive, domestic, and community responsibilities.

The transformation of food systems toward more efficient and sustainable production and longer value chains, in combination with shifts in diets toward greater consumption of processed foods and foods away from home, offers opportunities for women, but may also create new barriers to participation. Changes in the demand for different types of agricultural products, both food and nonfood, may affect women’s involvement in different value chains. Participation does not automatically translate to benefits: if participation occurs on terms that are unfavorable to women, they may not necessarily benefit from increased market inclusion. For example, as more people migrate to urban areas, women may increasingly work outside the home and families may rely more on the market, rather than own production, for food, which has implications for the health and nutritional status of household members. The consumption of processed, especially ultra-processed, foods with added sugar, fat, and sodium may lead to higher rates of chronic disease (Popkin, 1993), but minimally processed foods, such as homestyle processing that is common in many local value chains may reduce women’s workload with minimal change to nutritional value (Monteiro et al., 2019). Moreover, women’s work outside the home and in food systems specifically has implications for childcare, which may determine children’s diets and nutritional status, especially in contexts where the gendered division of responsibilities places childcare squarely within the woman’s domain. Women’s increased involvement in food systems is also associated with diets and nutrition outcomes for women themselves and other household members, although the direction of association and the pathways to impact are not yet fully understood. Moreover, there is a growing recognition that transforming food systems for inclusion means not just ensuring women’s participation and access to benefits but also their empowerment to make strategic life choices (Malapit et al., 2020a).

In this paper, we investigate the factors correlated with greater empowerment of women and gender equality within specific value chains that are embedded within food systems. We use primary data collected in Asia (Bangladesh, Philippines) and Africa (Benin, Malawi) to examine the relationships between women’s empowerment, gender equality, and participation in agricultural value chains. Following Kabeer (1999), we define women’s empowerment as the process by which people expand their ability to make strategic life choices, particularly in contexts in which this ability has previously been denied to them. In Kabeer’s definition, the ability to exercise choice encompasses three dimensions: resources (including not only access but also future claims to material, human, and social resources), agency (including processes of decision-making, negotiation, and even deception and manipulation), and achievements (well-being outcomes). We operationalize this definition of empowerment in the project-level Women’s Empowerment in Agriculture Index (WEAI) for Market Inclusion (pro-WEAI+MI). This metric, described in detail in Section 2.2, allows us to examine both absolute levels of empowerment of men and women and their relative empowerment within a household, and was specifically adapted for use in the context of value chains.

**Fig. 1:**
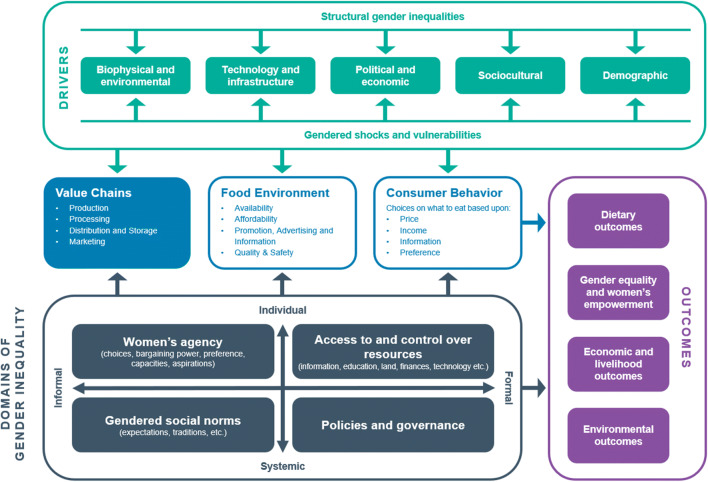
Gendered Food Systems Source: Reproduced with permission from Njuki et al. (2021), framework adapted from de Brauw et al. (2019)

We ground our approach in the Gendered Food Systems Framework (Njuki et al., 2021), itself an adaptation of the Food Systems Framework (De Brauw et al., 2019) (Fig. 1). The framework recognizes three distinct components of the food system: value chains, the food environment, and consumer behavior (center of Fig. 1). These three components are influenced by food systems drivers, which include biophysical and environmental, technological and infrastructural, political and economic, sociocultural, and demographic factors; factors that are themselves shaped by structural gender inequalities as well as gendered shocks and vulnerabilities (top of Fig. 1).

The three core food system components also interact with gender (in)equality, which is multidimensional. In this framework, there are two axes along which gender (in)equalities can vary: formal to informal, and individual to systemic (bottom left of Fig. 1). The three components of the food system, along with gender (in)equalities, drive food systems outcomes related to diets, gender equality and women’s empowerment, economic wellbeing and livelihoods, and the environment (right side of Fig. 1). Transforming food systems to empower women and enhance gender equality requires acknowledging the gender disparities along the corresponding value chains, identifying potential areas for these transformations to reach, benefit, and empower women (Malapit et al., 2020a; Johnson et al., 2018), and addressing gender inequalities across all these dimensions.

Njuki et al.’s (2021) review finds considerable evidence on the link between the consumer behavior component of food systems and women’s empowerment and gender equality, especially women’s roles in consumption and traditionally female activities, such as food preparation and feeding children. The existing evidence on the food environment component, which includes food availability and affordability and women’s access to markets, points to women’s relative poverty and limited freedom of movement as the primary factors constraining women’s empowerment and gender equality. This suggests that successful value chain-focused interventions could also expand women’s access to the food environment. However, evidence on value chains, the third component of food systems, and how they relate to women’s roles, women’s empowerment, and gender equality is scarce. This is not surprising, since attention to equity dimensions and development of gender-sensitive tools are a relatively recent development in food systems research (Getahun & Villanger, 2018; Said-Allsopp & Tallontire, 2015; Barrientos et al., 2003; Graef et al., 2018; Riisgaard et al., 2008; Rubin et al., 2009; Mayoux, 2012). Within the limited literature focused on traditional—or even high-value—crops and the engagement of women along the related value chains, some studies have identified pathways whereby food system commercialization can increase women’s involvement in specific activities (David, 2015; Djurfeldt et al., 2018; Forsythe et al., 2016; Getahun & Villanger, 2018; Handschuch & Wollni, 2013; Quisumbing et al., 2015). For example, in Northern Nigeria, David (2015) finds that the relatively flexible gender division of labor and women’s autonomy over decisions and income regarding personal farming plots were key factors that allowed women to successfully engage in income-generation through increased commercialization of sweet potato production, which was traditionally viewed as a “male” crop. In our framework, these are informal and individual factors. Similarly, female respondents in a case study of smallholder farmers in Myanmar (Herens et al., 2018) emphasized the ability of women to buy and inherit land and manage the farm as an important component of their engagement in crop commercialization. In our framework, these would qualify as formal and systemic factors affecting gender equality.

Interventions often aim to (i) enhance women’s roles in agricultural value chains where they already operate, for example, by increasing their involvement in specific nodes or stages of the value chain with the potential for value addition, such as processing or marketing, and (ii) expand opportunities for women to start operating within new value chains. When women are able to engage more directly or more extensively in these activities, either through formal employment or increased participation in high-value products or value-adding activities, some studies have found that they can increase their contributions to household incomes and resources (Handschuch & Wollni, 2013; Said-Allsopp & Tallontire, 2015; Quisumbing et al., 2015). While increasing opportunities for women’s engagement in food system commercialization can improve equality and empowerment and is often correlated with increasing their control over income and, relatedly, bargaining power within their households (Rubin et al., 2009; Getahun & Villanger, 2018), the link between market inclusion and women’s empowerment is not automatic.

In addition to identifying opportunities for increased engagement, studies have also highlighted the constraints that many women face when seeking employment, expanding their participation in value chains to activities such as processing and marketing, and increasing commercialization and market orientation (Ashby et al., 2008; Barrientos et al., 2003; Forsythe et al., 2016; Mayoux, 2012; Said-Allsopp & Tallontire, 2015). Across value chains and geographies, many women face similar barriers to greater engagement: social norms, asset constraints (especially land and money), and gender-inequitable employment conditions often limit the ways in which women engage with food systems (Ashby et al., 2008; Barrientos, Dolan, and Tallontire, 2003; David, 2015; Djurfeldt et al., 2018; Forsythe et al., 2016; Mayoux, 2012; Quisumbing et al., 2015).

Given this background, it is especially strategic to focus on how the value chains component of food systems relates to women’s empowerment and gender equality. Our analysis includes four countries with very different structural and social contexts. We expect the factors affecting women’s empowerment to vary quite widely both across and within countries, depending on the type of value chain considered. We begin by describing the data and methods used, including the pro-WEAI+MI indicators. We then discuss the learnings from the four country surveys and conclude with policy implications.

## Materials and methods

Value chain analysis is an approach that examines each step from production to consumption and provides an inclusive framework for characterizing many dimensions of a food system, including agricultural production, food supply, and food affordability (Gelli et al., 2015). Although a food system can comprise many value chains, the focus on specific value chains (commodities) can help identify specific characteristics of different value chains or nodes of a value chain that are differentially associated with women’s empowerment and gender equality. We use value chain analysis to examine the correlates of women’s and men’s empowerment and gender equality using data from the four countries where the pro-WEAI+MI was piloted.

### Data and context

IFPRI piloted the pro-WEAI+MI in two countries in Asia (Bangladesh and the Philippines), and two countries in Africa (Benin and Malawi), all with very different socio-cultural contexts. The pilot studies originally referred to the instrument as WEAI for Value Chains (WEAI4VC). This instrument is now called pro-WEAI for Market Inclusion (pro-WEAI+MI), to emphasize that it collects the core pro-WEAI module together with complementary information related to market inclusion.

The pro-WEAI+MI adapts the pro-WEAI approach, which focuses primarily on agricultural production, to account for men and women who are active in processing and marketing nodes of the value chains (Malapit et al., 2019). We computed pro-WEAI metrics based on the data collected in all four countries. Sampling for these studies varied according to the purpose of the study and is not nationally representative. The Bangladesh (Raghunathan et al., 2021; Ahmed et al., 2018) and Philippines (Malapit et al., 2020a) studies were conducted as standalone pilots to develop pro-WEAI+MI and were not associated with a gender-sensitive intervention. The Benin and Malawi studies were conducted as part of mixed-methods evaluations of two gender-sensitive vocational training programs (Agricultural Technical and Vocational Education Training for Women Program, ATVET4W) (Heckert et al., 2021; Ragasa et al., 2021). We do not use the intervention design to make any inferences about program treatment effects in the analysis presented in this paper.

**Fig. 2:**
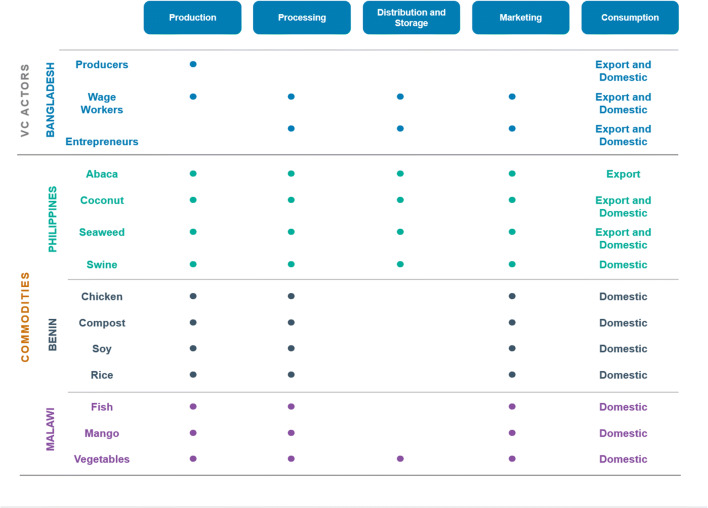
Summary of the value chains studied in each country

Figure 2 presents a graphical summary of the specific value chains and nodes thereof for each of the four countries in our sample. These are arranged by country (rows) and along the nodes of the value chains from production to consumption (columns). The design of the Bangladesh study (top panel) differs from the others in that it captures different actors across different nodes of the value chain. The other country studies focused on specific commodity value chains, which differ across countries, and the nodes within those value chains. Although each commodity may be purchased by consumers in different destinations (domestic or international), we do not focus on this aspect in our analysis.

#### Bangladesh

The Bangladesh survey data were collected from May to July 2017, covering 1200 households in the Feed the Future Zone of Influence (FTF ZOI) in southwestern Bangladesh (see Ahmed et al., 2018 for details on sampling). Sample households were selected to have equally sized groups across three livelihood types (agricultural production, agricultural entrepreneurship, and agricultural wage employment). The livelihood type was determined at the household level, thus a woman who is a producer in an entrepreneur household is classified as an entrepreneur. Detailed individual and household surveys were administered to the primary male and female decision makers from the final sample of 1200 households by interviewers of the same sex.

A qualitative study was also conducted between August and October 2017 (Rubin et al., 2018). It included a total of 102 interviews with a subset of interviewees from the quantitative survey across the same three livelihood types; key informant interviews with market traders, community leaders, and government officials knowledgeable about the study communities; and focus group discussions with four to five male and female participants according to livelihood type. Participants were interviewed by interviewers of the same sex. The qualitative study examined respondents’ engagement with agricultural value chains, their sources of livelihood, barriers to women’s participation in these livelihood activities, and respondents’ understanding of the meaning of ‘empowerment.’

#### Philippines

The Philippine study focused on four value chains—abaca, coconut, seaweed, and swine—that are commodities with high potential for growth. The survey data were collected from March to August 2017 using a purposive sampling design focusing on top-producing provinces and villages in the Bicol and Visayas regions of the Philippines to ensure sufficient respondents for each value chain and node (see Malapit et al., 2020b for details). The target sample size for each province-commodity group was 200 households, totaling 400 households per commodity and 1600 households for the entire survey; in practice, 1264 households and 2811 individuals were interviewed. A brief formative qualitative study was conducted to inform the study design and identify key areas of inquiry related to empowerment and gender norms. After the survey was completed, more in-depth qualitative data were collected from September to December 2017 to provide insights into some of the key results and patterns emerging from the pro-WEAI+MI analysis. The second round of qualitative work drew on qualitative protocols developed for pro-WEAI in combination with gender and agricultural value chain approaches (Rubin et al., 2009; Meinzen-Dick et al., 2019) to address specific concerns related to participation and benefits at different nodes of the coconut and seaweed value chains. Interviews were conducted with a subset of male and female respondents from the quantitative survey.

#### Benin

In Benin and Malawi, the studies were conducted as part of impact assessments of two Agricultural Technical Vocational Education and Training (ATVET) for Women programs, which were being promoted by the African Union Commission (AUC) and African Union Development Agency-New Partnership for Africa’s Development (AUDA-NEPAD) with the support of Deutsche Gesellschaft für Internationale Zusammenarbeit (GIZ). ATVET for Women (ATVET4Women) provides training for women in selected occupations in high-priority value chains. The Benin intervention focused on training women producers in rice, soy, chicken, and compost (Heckert et al., 2021). A survey was conducted from August to September 2019 in Donga, Collines, and Atakora departments in the north and Atlantique and Ouémé departments in the South. The sample included both program trainees as well as a comparison group. Both were selected through similar approaches, and thus all respondents were active in one of the four target value chains. The total sample included 879 households (242 beneficiary, and 637 non-beneficiary), for a total of 879 women and 589 men. The survey included the pro-WEAI+MI instruments, plus several new modules that were being piloted, all of which benefited from cognitive interviewing to ensure questions were well understood by respondents. The qualitative study was conducted in the same areas as the survey from November 2019 to January 2020. A total of 58 interviews were conducted with program beneficiaries, husbands of beneficiaries, and non-beneficiaries, all selected from the quantitative sample and other value chain actors, who were input suppliers, extension agents, credit providers, local traders, program trainers, and agro-processing center managers. Data collection aimed to understand issues around the gender dimensions of participation in, benefits from, and empowerment at different nodes of the value chain.

#### Malawi

The Malawi study drew on the non-formal training component of the ATVET4Women program in Malawi, which jointly trained couples, aiming to increase production and profits from involvement in fish, mango, and vegetable value chains (Ragasa et al., 2021). A survey was conducted from September to October 2019. The sample came from five districts spread across Malawi, included program graduates and a comparison group, and covered women and men from 544 households for a total of 542 women and 395 men. As with Benin, the pro-WEAI+MI survey content benefited from cognitive interviewing and covered the same content. The Malawi qualitative study was conducted from November 2019 to February 2020. A total of 51 interviews were conducted with women producers who graduated from the program, their husbands, women producers who were not program trainees, and women graduates who were entrepreneurs (processors and traders), all selected from the quantitative sample; program instructors; an agricultural extension officer; and stakeholders (private, government, and NGO sectors). Data collection focused on program benefits, market integration, and local understandings of empowerment.

### The pro-WEAI for market inclusion

Our measures of empowerment and gender equality are drawn from the pro-WEAI+MI. This instrument is based on the WEAI, an internationally-recognized approach for measuring women’s empowerment in the context of agricultural production, originally developed by the International Food Policy Research Institute (IFPRI), the Oxford Poverty and Human Development Initiative (OPHI), and United States Agency for International Development (USAID) (Alkire et al., 2013). In response to demands from implementers and other partners, the WEAI was adapted to suit the needs of various types of agricultural development projects, leading to the development of the project-level WEAI, or pro-WEAI (Malapit et al., 2019).

The pro-WEAI includes 12 indicators mapped to three domains reflecting three different types of agency: intrinsic agency (power within), instrumental agency (power to), and collective agency (power with). An individual is deemed adequate on a given indicator if they meet a certain threshold (see Appendix 1 for definitions of the indicators) and is defined as empowered if they have adequate achievements in 9 out of the 12 indicators. The pro-WEAI consists of two sub-indices – the Three Domains of Empowerment, or 3DE, which measures men’s and women’s performance on the 12 indicators, and the Gender Parity Index, or GPI, which captures women’s achievements in the three domains relative to those of the man in the same household. The latter is only calculated for households with both men and women respondents (dual-headed households or DHHs). In addition to the quantitative measures, the pro-WEAI also includes qualitative tools to help projects understand local definitions of empowerment.

The pro-WEAI+MI, the measure used in this paper, uses the pro-WEAI as its starting point, and adds components to capture empowerment across activities along the relevant value chains. Because the tool was being developed while the pilots were ongoing, some indicators are calculated differently in some countries. Additionally, the Benin and Malawi studies did not include the *frequency of visiting important places* indicator and empowerment is calculated as 8 out of 11 indicators.

### Empirical specification

We use regression analysis to examine the factors associated with different empowerment outcomes at the individual and household level. At the individual level, we are concerned with empowerment of individuals *i (Empowerment)*; at the household level, we analyze the difference between empowerment outcomes of the primary man and woman, which we broadly define as intrahousehold inequality of household *j (Intrahousehold Inequality)* (for dual-headed households only).

#### Individual regressions

We analyze correlates of individual empowerment:

1 $$ {Empowerment}_i={\boldsymbol{\beta}}_{ind}^{\prime }{\boldsymbol{X}}_i+{\varepsilon}_i $$

Where ***X***_*i*_ are individual- and household-level factors explaining *Empowerment;* ${\boldsymbol{\beta}}_{ind}^{\prime }$ is a vector of coefficients to be estimated; and *ε*_*i*_ are error terms to be estimated. Individual-level regressions are estimated separately for women and men. We use two indicators of overall empowerment:

1. whether the individual is empowered or not (a binary variable, 0/1);
2. empowerment score based on 3DE (continuous variable, from 0 to 1).

#### Household level regressions

We also analyze the correlates of intrahousehold inequality, measured at the household level:

2 $$ {Intrahousehold\ Inequality}_j={\boldsymbol{\beta}}_{hh}^{\prime }{\boldsymbol{Z}}_j+{\varepsilon}_j $$

Where ***Z***_*j*_ are household-level factors explaining *Intrahousehold Inequality*, which differ slightly across study sites; ${\boldsymbol{\beta}}_{hh}^{\prime }$ are coefficients to be estimated; and *ε*_*j*_ are error terms to be estimated. Household-level regressions are estimated for dual-headed households only.

We measure intrahousehold inequality as the difference between men’s and women’s empowerment scores (a continuous variable, from −1 to 1). A positive inequality score means that men are more empowered than women in the household, while a negative inequality score means that women are more empowered than men in the household. If gender equality is a desired outcome, the interpretation of regression coefficients using a continuous intrahousehold inequality variable would be ambiguous. To avoid this, we construct a categorical variable defined as: (i) whether the man is more empowered than the woman, (ii) whether the woman is more empowered than the man, or (iii) whether the man and woman achieve similar levels of empowerment. Using multinomial logit regressions, we estimate the likelihood that a man (or woman) is more empowered, relative to the excluded category where the man and woman are equally empowered.

## Results

### Descriptive statistics

Across all countries, women respondents tend to be younger than men, although the magnitude of the age difference varies (Table 1). In Benin and Malawi, men respondents have more years of schooling, whereas in the Philippines and Bangladesh women complete more years of schooling than men. This pattern is common in the Philippines but is a relatively recent phenomenon in Bangladesh owing to policies encouraging girls’ education. Most of our respondents are married and live in dual-adult households, although about a fifth of respondents in Benin and the Philippines live in woman-only households.

**Table 1:** Characteristics of women and men respondents: Bangladesh, Philippines, Benin, Malawi

	Bangladesh		Philippines		Benin		Malawi	
	Women	Men	Women	Men	Women	Men	Women	Men
Number of observations	1144	1063	1606	1183	703	497	510	363
Age	36.4	43.9	49.0	49.8	37.3	43.3	38.0	42.7
Years of schooling	5.1	4.8	6.7	5.6	2.6	3.8	6.3	7.6
Household size	4.5	4.6	4.6	4.8	5.9	6.1	5.7	5.7
Marital status (% married)	96.9	97.2	74.2	86.6	90.0	98.4	86.5	99.4
Lives in dual-headed household (%)	93.3	100.0	78.6	100.0	81.0	100.0	91.4	100.0
Lives in woman-only household (%)	6.7	n/a	21.4	n/a	19.0	n/a	8.6	n/a

Notes: n/a: not applicable

Table 2 shows the 3DE score, the GPI, and the pro-WEAI score. Because our samples were drawn purposively, these results should not be interpreted as representative of the empowerment status of women and men in these countries but may be indicative of the types of households targeted for the interventions or involved in the target value chains. Except for the Philippines, women’s 3DE scores (scores across the three domains of empowerment) are consistently lower than men’s, although levels vary. Women in the Malawi sample have the highest 3DE score, followed by the Philippines, Benin, and Bangladesh, in that order. More than 90% of Bangladeshi women in our sample are disempowered, while about two-thirds of the women in the Philippines and Benin samples have not achieved empowerment. In contrast, only 27% of the women in the Malawi sample have not achieved empowerment. The GPI is highest in the Malawi sample, followed by the Philippine and Benin samples, with Bangladesh showing the least gender parity. Pro-WEAI scores range from 0.53 in Bangladesh to 0.89 in Malawi.

**Table 2:** Empowerment status, average empowerment scores, and gender parity: Bangladesh, Philippines, Benin, and Malawi

	Bangladesh		Philippines		Benin		Malawi	
	Women	Men	Women	Men	Women	Men	Women	Men
Number of observations	1144	1063	1461	1061	703	497	510	363
**3DE Score**	0.54	0.75	0.73	0.73	0.66	0.83	0.88	0.93
Disempowerment score (1-3DE)	0.46	0.25	0.27	0.27	0.34	0.17	0.11	0.06
% achieving empowerment	8	26	33	33	31	61	73	85
% not achieving empowerment	92	74	67	67	69	39	27	15
Mean adequacy score for not yet empowered	0.50	0.66	0.60	0.59	0.50	0.56	0.58	0.57
Mean disempowerment score (1-adequacy) for not yet empowered	0.50	0.34	0.40	0.41	0.50	0.44	0.42	0.41
Number of dual-adult households	2130		1061		577		466	
**Gender parity index (GPI)**	0.49		0.92		0.82		0.95	
% achieving gender parity	2		65		42		73	
% not achieving gender parity	98		35		58		27	
Average empowerment gap	0.51		0.23		0.32		0.20	
**Pro-WEAI score**	0.53		0.75		0.67		0.89	
Households in which man is more empowered (% of total)	32		20		48		34	
Households in which woman is more empowered (% of total)	5		21		10		12	

Source: Authors’ calculations.

### Correlates of empowerment and gender equality

Before turning to the regression analysis, we note a few additional details. First, the upper part of each regression table (Tables 3-10) contains the key variables of interest—those related to value chain and market participation (Bangladesh, Philippines) and value chain, training, and market outcomes (Benin, Malawi). Second, we use principal component analysis to construct an asset index from information on household assets (including indicators of quality of dwelling, ownership of productive equipment, land, and livestock) and divide households into quintiles based on their score on that index. Because asset lists vary across countries, the wealth quintiles referred to below are country and survey specific. Third, the regressions to follow are intended to capture correlations, not causation. In presenting our results, we use “correlations” and “associations” interchangeably because they do not imply causality.

**Table 3:** Correlates of women’s and men’s empowerment, Bangladesh

	Whether empowered (=1 if empowered)^a^		Empowerment score (continuous)^b^	
	Women	Men	Women	Men
**Value chain and market participation characteristics**				
*Household type (ref. = household is a producer)*				
Household is an entrepreneur (=1)	−0.049***	0.058*	−0.027***	0.014***
	(0.014)	(0.032)	(0.005)	(0.003)
Household is a wage earner (=1)	−0.083***	−0.018	−0.042***	0.009***
	(0.016)	(0.030)	(0.004)	(0.003)
**Individual and household characteristics**				
Respondent is in a woman-only household (WOH)	0.107*		0.007***	
	(0.057)		(0.001)	
Highest educational level of respondent	−0.004	0.012**	0.019*	0.015***
	(0.006)	(0.006)	(0.010)	(0.005)
Married (=1)	0.015	0.047	0.012	0.039*
	(0.023)	(0.055)	(0.030)	(0.021)
Age of respondent (years)	0.006	0.003	0.636***	−0.022
	(0.004)	(0.004)	(0.086)	(0.072)
Age squared	−0.000*	−0.000	−0.291***	0.016
	(0.000)	(0.000)	(0.042)	(0.036)
Household size	−0.002	−0.009	−0.020	−0.012
	(0.005)	(0.006)	(0.013)	(0.009)
Household received cash assistance/transfer (=1)	−0.011	0.014	0.001	0.002
	(0.014)	(0.025)	(0.003)	(0.002)
Household received in-kind assistance/transfer (=1)	−0.014	0.059*	0.003	0.003
	(0.013)	(0.030)	(0.003)	(0.002)
*Asset/wealth quintile (ref. = poorest)*				
Quintile 2	−0.006	−0.006	0.002	0.004
	(0.020)	(0.047)	(0.003)	(0.002)
Quintile 3	−0.007	0.012	−0.002	0.005*
	(0.017)	(0.047)	(0.004)	(0.003)
Quintile 4	−0.016	−0.026	−0.005	0.005*
	(0.018)	(0.042)	(0.004)	(0.003)
Quintile 5 (Richest)	−0.056***	0.081	−0.011***	0.013***
	(0.017)	(0.055)	(0.004)	(0.003)
Observations	1144	1063	1144	1063
Pseudo R-squared	0.192	0.065	0.024	0.005

Source: Raghunathan et al. (2021); ^a^Estimated using logit regression. ^b^Estimated using fractional regression

Note: Marginal effects reported, standard errors in parentheses. (=1) represents dummy variables and coefficients denote the effect of a discrete change in the dummy variable from 0 to 1. Asset index was calculated using principal components analysis based on roof material, floor material, number of bedrooms, improved toilet, access to electricity, improved cook fuel source, dwelling in excellent state, and ownership of land, large livestock, fishing equipment, mechanized farm equipment, inventory/stock business, non-agricultural land, mechanized means of transport, shop facility, and storage facility

* p < 0.10, ** p < 0.05, ***p < 0.01

**Table 4:** Correlates of intrahousehold measures of empowerment (dual-headed households only), Bangladesh

	Gender parity achieved=1^a^	Whether man more empowered (=1)^b^	Whether woman more empowered (=1)^b^
**Value chain and market participation characteristics**			
*Household type (ref. = household is a producer)*			
Household is an entrepreneur (=1)	−0.207***	0.212***	−0.086***
	(0.040)	(0.035)	(0.020)
Household is a wage earner (=1)	−0.266***	0.357***	−0.151***
	(0.037)	(0.034)	(0.028)
**Individual and household characteristics**			
Highest educational level, male respondent	−0.003	0.003	−0.006
	(0.009)	(0.009)	(0.005)
Highest educational level, female respondent	0.001	−0.008	0.001
	(0.011)	(0.011)	(0.006)
Married (=1), male respondent	0.124	−0.002	0.044
	(0.102)	(0.141)	(0.088)
Married (=1), female respondent	0.100	−0.141	1.007***
	(0.137)	(0.192)	(0.161)
Age (years), male respondent	−0.000	0.006	−0.006
	(0.013)	(0.013)	(0.008)
Age (years), female respondent	0.031*	−0.046***	0.010
	(0.017)	(0.014)	(0.009)
Age squared, male respondent	−0.000	−0.000	0.000
	(0.000)	(0.000)	(0.000)
Age squared, female respondent	−0.000	0.001***	−0.000
	(0.000)	(0.000)	(0.000)
Household size	−0.001	−0.002	−0.002
	(0.008)	(0.008)	(0.005)
Household received cash assistance/transfer (=1)	0.036	−0.017	−0.037*
	(0.033)	(0.033)	(0.020)
Household received in-kind assistance/transfer (=1)	−0.038	−0.039	−0.005
	(0.036)	(0.037)	(0.020)
*Asset/wealth quintile (ref = poorest)*			
Quintile 2	−0.016	0.003	−0.051*
	(0.038)	(0.049)	(0.027)
Quintile 3	−0.065	0.055	−0.040
	(0.042)	(0.051)	(0.027)
Quintile 4	−0.092**	0.119**	−0.037
	(0.042)	(0.055)	(0.027)
Quintile 5 (Richest)	−0.201***	0.265***	−0.055
	(0.044)	(0.064)	(0.035)
Constant			
Observations	1069	1059	1059
Pseudo R-squared	0.121	0.127	0.127
*Households in which empowerment scores are equal (% of total)*	*426 (40.19)*		
*Households in which man is more empowered (% of total)*	*559 (52.74)*		
*Households in which woman is more empowered (% of total)*	*75 (7.08)*		

^a^Gender parity is defined as the woman being equally or more empowered than the primary male adult in the household; estimated using logit

^b^Estimated using multinomial logit, with base defined as households where woman and man are equally empowered

Note: Marginal effects reported, standard errors in parentheses. (=1) represents dummy variables and coefficients denote the effect of a discrete change in the dummy variable from 0 to 1

* p < 0.10, ** p < 0.05, ***p < 0.01

See notes to Table 3

**Table 5:** Correlates of women’s and men’s empowerment, Philippines

	Whether empowered (=1)^a^		Empowerment score (continuous)^b^	
	Women	Men	Women	Men
**Value chain and market participation characteristics**				
*VC main activity (reference = production)*				
Processing	−0.043	0.010	−0.023**	−0.013
	(0.029)	(0.035)	(0.011)	(0.014)
Trading	−0.006	−0.082**	−0.002	−0.009
	(0.035)	(0.041)	(0.009)	(0.010)
*Main VC (reference = seaweed)*				
Abaca	−0.076**	−0.099**	−0.037***	−0.053***
	(0.035)	(0.040)	(0.013)	(0.016)
Coconut	−0.138***	−0.212***	−0.081***	−0.085***
	(0.034)	(0.037)	(0.012)	(0.016)
Swine	−0.134***	−0.140***	−0.057***	−0.046***
	(0.034)	(0.041)	(0.013)	(0.015)
*Other market participation*				
Participates in non-farm activities (=1)	−0.001	0.004	0.004	−0.014
	(0.027)	(0.034)	(0.012)	(0.014)
Participates in wage employment (=1)	0.008	−0.077**	0.001	−0.036*
	(0.029)	(0.031)	(0.011)	(0.019)
**Other individual and household characteristics**				
Respondent is in a woman-only household (WOH)	−0.022		0.003	
	(0.039)		(0.011)	
Highest educational level of respondent	0.013*	0.024***	0.090**	0.135***
	(0.007)	(0.008)	(0.038)	(0.041)
Married (=1)	0.067*	0.065	0.081**	0.122**
	(0.036)	(0.045)	(0.036)	(0.052)
Age of respondent (years)	0.001	0.001	0.118*	0.157*
	(0.001)	(0.001)	(0.070)	(0.091)
Access to extension	0.050*	0.123***	0.039***	0.074***
(=1)	(0.027)	(0.032)	(0.013)	(0.017)
Access to community	0.060*	0.045	0.133***	0.128***
programs (=1)	(0.031)	(0.035)	(0.031)	(0.033)
*Asset/wealth quintile*^*†*^*(reference = poorest)*				
Quintile 2	−0.013	−0.070	−0.013	0.003
	(0.041)	(0.045)	(0.011)	(0.013)
Quintile 3	0.040	−0.014	−0.001	0.007
	(0.043)	(0.049)	(0.011)	(0.013)
Quintile 4	0.043	−0.050	0.006	0.001
	(0.043)	(0.048)	(0.011)	(0.013)
Quintile 5	0.094**	0.013	0.003	−0.003
	(0.046)	(0.053)	(0.012)	(0.013)
**Observations**	**1410**	**1041**	**1410**	**1041**
**Pseudo R-squared**	**0.037**	**0.064**	**0.13**	**0.11**

Source: Malapit et al. (2020) ^a^Estimated using logit regression ^b^Estimated using fractional regression

Marginal effects reported, standard errors in parentheses. (=1) represents dummy variables and coefficients denote the effect of a discrete change in the dummy variable from 0 to 1. Estimates using municipality and province fixed effects were largely consistent. Asset index was calculated using principal components analysis based on roof material, floor material, people per sleeping room, state of dwelling, type of toilet, source of water and drinking water, electricity, source of cooking fuel, and ownership of land, boats, fishponds, farm equipment, business equipment, consumer durables, cell phones, houses, and means of transportation

* p < 0.05, ** p < 0.01, *** p < 0.001

**Table 6:** Correlates of intrahousehold measures of empowerment, (dual-adult households only), Philippines

	Whether gender parity is achieved (=1) ^a^	Whether man is more empowered (=1)^b^	Whether woman is more empowered (=1)^b^
**Value chain and market participation characteristics**			
*Man’s participation in different nodes of the VC (reference = production)*			
Processing	0.032	−0.358	−0.564
	(0.070)	(0.599)	(0.550)
Trading	−0.269**	1.416**	0.049
	(0.126)	(0.629)	(0.584)
*Woman’s participation in different nodes of the VC (reference = production)*			
Processing	−0.039	0.352	0.399
	(0.077)	(0.599)	(0.551)
Trading	0.181***	−1.517**	−0.164
	(0.053)	(0.634)	(0.574)
*Main VC (reference = seaweed)*			
Abaca	−0.051	0.374*	0.283
	(0.036)	(0.222)	(0.220)
Coconut	0.021	−0.143	−0.008
	(0.035)	(0.243)	(0.236)
Swine	−0.045	0.278	0.019
	(0.038)	(0.237)	(0.233)
*Other market participation*			
Man respondent participated in non-farm activities (=1)	0.058*	−0.430*	−0.097
	(0.034)	(0.243)	(0.230)
Woman respondent participated in non-farm activities (=1)	−0.013	0.183	0.291
	(0.035)	(0.230)	(0.221)
Man respondent participated in wage employment (=1)	0.046*	−0.372**	−0.259
	(0.025)	(0.170)	(0.166)
Woman respondent participated in wage employment (=1)	0.038	−0.217	0.071
	(0.027)	(0.190)	(0.176)
**Individual and household characteristics**			
Household size	0.003	−0.027	−0.034
	(0.006)	(0.040)	(0.039)
Highest educational level of man respondent	−0.008	0.034	−0.092*
	(0.008)	(0.050)	(0.051)
Highest educational level of woman respondent	0.015*	−0.088*	0.012
	(0.008)	(0.053)	(0.050)
Age of man respondent (years)	−0.002	0.012	−0.014
	(0.002)	(0.012)	(0.011)
Age of woman respondent (years)	0.003*	−0.020*	−0.004
	(0.002)	(0.012)	(0.011)
Man respondent has access to extension services (=1)	−0.066**	0.281*	−0.521***
	(0.027)	(0.167)	(0.176)
Woman respondent has access to extension services (=1)	0.052**	−0.320*	0.095
	(0.025)	(0.177)	(0.171)
Man respondent has access to community programs (=1)	0.030	−0.268	−0.322*
	(0.030)	(0.188)	(0.180)
Woman respondent has access to community programs (=1)	−0.063**	0.400*	−0.143
	(0.028)	(0.213)	(0.199)
*Asset/wealth quintile* ^*†*^ *(reference = poorest quintile)*			
Asset quintile 2	−0.025	0.174	0.097
	(0.039)	(0.249)	(0.237)
Asset quintile 3	−0.064	0.466*	0.306
	(0.043)	(0.258)	(0.249)
Asset quintile 4	−0.043	0.287	0.109
	(0.042)	(0.264)	(0.257)
Asset quintile 5 (richest)	−0.027	0.131	−0.133
	(0.045)	(0.283)	(0.278)
Constant		−0.491	0.688
		(0.613)	(0.587)
**Observations (total number of households)**	**1134**	**1134**	
*Households in which empowerment scores are equal (% of total)*	664 (58.6)		
*Households in which man is more empowered (% of total)*		230 (20.2)	
*Households in which woman is more empowered (% of total)*			240 (21.2)
**Pseudo R-squared**	**0.036**	**0.036**	

Source: Malapit et al. (2020)

^a^Gender parity is defined as the woman being equally or more empowered than the main man in the household; estimated using logistic regression

^b^Estimated using multinomial logit, with base defined as households where woman and man are equally empowered. Marginal effects reported, standard errors in parentheses. (=1) represents dummy variables and coefficients denote the effect of a discrete change in the dummy variable from 0 to 1

* p < 0.10, ** p < 0.05, ***p < 0.01. p < 0.01

See additional notes to Table 4

**Table 7:** Correlates of women’s and men’s empowerment, Benin

	Whether empowered (=1)^a^		Empowerment score (continuous)^b^	
	Women	Men	Women	Men
**Value chains, training, and market outcomes**				
*Household type (reference = Rice)*				
Soy	−0.060	−0.045	−0.008	0.001
	(0.065)	(0.120)	(0.009)	(0.009)
Compost	0.049	0.283***	0.004	0.006***
	(0.096)	(0.076)	(0.002)	(0.002)
Poultry	−0.056	0.106	−0.002	0.008
	(0.085)	(0.113)	(0.006)	(0.006)
Received ATVET4W training? (=1)	0.064	0.276***	0.003	0.002**
	(0.051)	(0.081)	(0.004)	(0.001)
**Individual and household characteristics**				
Respondent is in a woman-only household (WOH)	0.154***		0.012***	
	(0.057)		(0.003)	
Highest educational level of respondent	0.072*	0.017	0.040**	−0.003
	(0.043)	(0.035)	(0.016)	(0.013)
Married (=1)	−0.001	0.253	0.005	0.040
	(0.080)	(0.237)	(0.023)	(0.047)
Age of respondent (years)	0.007***	0.001	0.097***	0.020
	(0.002)	(0.002)	(0.023)	(0.026)
Severely Food Insecure (FIES)	0.031	−0.055	0.002	−0.003
	(0.051)	(0.068)	(0.002)	(0.003)
Household size	−0.001	0.003	−0.016	0.024*
	(0.007)	(0.008)	(0.013)	(0.014)
*Asset/Wealth Quintile (reference = poorest)*				
Quintile 2	0.094	0.185**	0.008*	0.013***
	(0.080)	(0.093)	(0.005)	(0.004)
Quintile 3	0.057	0.168*	0.007	0.013***
	(0.085)	(0.100)	(0.005)	(0.004)
Quintile 4	0.174**	0.207**	0.013***	0.018***
	(0.076)	(0.084)	(0.004)	(0.006)
Quintile 5 (Richest)	0.178**	0.253***	0.013***	0.024***
	(0.090)	(0.089)	(0.005)	(0.005)
**Observations**	702	497	703	497
Pseudo R-squared	0.110	0.120	0.017	0.016

Source: Authors’ calculations

^a^Estimated using logit regression

^b^Estimated using fractional regression. Marginal effects reported, standard errors in parentheses. (=1) represents dummy variables and coefficients denote the effect of a discrete change in the dummy variable from 0 to 1. Asset index was calculated using principal components analysis based on roof material, floor material, number of bedrooms, improved toilet, access to electricity, improved cook fuel source, dwelling in excellent state, and ownership of land, large livestock, fishing equipment, mechanized farm equipment, inventory/stock business, non-agricultural land, mechanized means of transport, shop facility, and storage facility

* p < 0.10, ** p < 0.05, ***p < 0.01

**Table 8:** Correlates of intrahousehold inequality (dual-headed households), Benin

	Whether gender parity is achieved (=1) ^a^	Whether man is more empowered (=1)^b^	Whether woman is more empowered (=1)^b^
**Value chains, training, and market outcomes**			
*Household type (reference = rice)*			
Soy	0.146**	−0.079	−0.067*
	(0.065)	(0.065)	(0.038)
Compost	0.078	0.048	−0.126**
	(0.111)	(0.111)	(0.060)
Poultry	0.021	0.065	−0.086*
	(0.086)	(0.084)	(0.049)
Received ATVET4W training (=1)	−0.069	−0.029	0.098**
	(0.084)	(0.082)	(0.048)
**Individual and household characteristics**			
Highest educational level, male respondent	−0.052	0.015	0.037**
	(0.037)	(0.036)	(0.018)
Highest educational level, female respondent	0.123**	−0.118*	−0.005
	(0.060)	(0.062)	(0.030)
Married status (=1), male respondent	−0.011	0.081	−0.070
	(0.229)	(0.234)	(0.133)
Married status (=1), female respondent	0.112	−0.156	0.044
	(0.182)	(0.174)	(0.113)
Age (years), male respondent	−0.006*	0.007**	−0.001
	(0.004)	(0.004)	(0.002)
Age (years), female respondent	0.005	−0.008**	0.003
	(0.004)	(0.004)	(0.002)
Severely Food Insecure (FIES)	−0.009	−0.021	0.030
	(0.061)	(0.059)	(0.037)
Household size	0.007	−0.000	−0.006
	(0.009)	(0.009)	(0.006)
*Asset/wealth quintile (reference = poorest)*			
Quintile 2	0.002	0.045	−0.047
	(0.125)	(0.125)	(0.063)
Quintile 3	−0.017	0.116	−0.098
	(0.125)	(0.125)	(0.063)
Quintile 4	0.094	−0.027	−0.067
	(0.120)	(0.120)	(0.059)
Quintile 5 (Richest)	0.063	0.058	−0.121**
	(0.118)	(0.119)	(0.059)
**Observations**	470	470	470
**Pseudo R-squared**	0.115	0.115	0.115
*Households in which empowerment scores are equal (% of total)*	194 (41.3)		
*Households in which man is more empowered (% of total)*		228 (48.5)	
*Households in which woman is more empowered (% of total)*			48 (10.2)

*Source:* Authors’ calculations

^a^Gender parity is defined as the woman being equally or more empowered than the main man in the household; estimated using logistic regression

^b^Estimated using multinomial logit, with base defined as households where woman and man are equally empowered. Marginal effects reported, standard errors in parentheses. (=1) represents dummy variables and coefficients denote the effect of a discrete change in the dummy variable from 0 to 1

* p < 0.10, ** p < 0.05, ***p < 0.01

FIES=Food Insecurity Access Scale

See additional notes for Table 7

**Table 9:** Correlates of women’s and men’s empowerment, Malawi

	Whether empowered (=1)^a^		Empowerment score (continuous)^b^	
	Women	Men	Women	Men
**Value chains, training, and market outcomes**				
*Household main value chain (reference = other commodities)*				
Mango producers (=1)	−0.086	−0.128	−0.008	−0.010
	(0.128)	(0.111)	(0.009)	(0.007)
Vegetable producers (=1)	0.004	0.062	0.002	0.000
	(0.050)	(0.041)	(0.007)	(0.008)
Ever received ATVET4W (=1)	0.042	−0.033	0.002	0.001
	(0.048)	(0.039)	(0.003)	(0.005)
Received other agricultural training (=1)	0.134***	0.099***	0.017***	0.013***
	(0.042)	(0.034)	(0.005)	(0.004)
**Individual and household characteristics**				
Respondent is in a woman-only household (WOH)	0.119		0.003*	
	(0.080)		(0.002)	
Highest educational level of respondent	0.078*	0.097***	0.043***	0.056***
	(0.040)	(0.027)	(0.015)	(0.014)
Married (=1)	0.023		−0.014	−0.038
	(0.090)		(0.021)	(0.041)
Age of respondent (years)	0.003	0.001	0.046*	−0.013
	(0.002)	(0.001)	(0.025)	(0.022)
Severely Food Insecure (FIES)	−0.033	−0.039	0.000	−0.002
	(0.057)	(0.041)	(0.004)	(0.005)
Household size	0.000	−0.007	−0.017	0.011
	(0.010)	(0.007)	(0.020)	(0.016)
*Asset/Wealth Quintile (reference = poorest)*				
Quintile 2	0.021	0.005	0.002	−0.003
	(0.057)	(0.040)	(0.003)	(0.004)
Quintile 3	0.090*	−0.008	0.005	−0.005
	(0.051)	(0.055)	(0.004)	(0.004)
Quintile 4	0.043	−0.014	0.002	−0.001
	(0.060)	(0.050)	(0.003)	(0.004)
Quintile 5 (Richest)	0.064	0.080**	0.005	0.000
	(0.060)	(0.039)	(0.004)	(0.003)
**Observations**	510	353	510	361
**Pseudo R-squared**	0.066	0.199	0.011	0.018

*Source*: Authors’ calculations

^a^Estimated using logit regression

^b^Estimated using fractional regression. Marginal effects reported, standard errors in parentheses. (=1) represents dummy variables and coefficients denote the effect of a discrete change in the dummy variable from 0 to 1. Asset index was calculated using principal components analysis based on roof material, floor material, number of bedrooms, improved toilet, access to electricity, improved cook fuel source, dwelling in excellent state, and ownership of land, large livestock, fishing equipment, mechanized farm equipment, inventory/stock business, non-agricultural land, mechanized means of transport, shop facility, and storage facility

* p < 0.10, ** p < 0.05, ***p < 0.01

+ All but 3 men in sample are married, so this variable was omitted

FIES=Food Insecurity Access Scale

**Table 10:** Correlates of intrahousehold inequality (dual-headed households), Malawi

	Whether gender parity is achieved (=1) ^a^	Whether man is more empowered (=1)^b^	Whether woman is more empowered (=1)^b^
**Value chains, training, and market outcomes**			
*Household main value chain (reference = other commodities)*			
Mango producers (=1)	0.188	−0.150	−0.038
	(0.171)	(0.154)	(0.146)
Vegetable producers (=1)	0.055	−0.022	−0.033
	(0.073)	(0.069)	(0.046)
Ever received ATVET4W (=1)	0.068	−0.274**	0.206***
	(0.134)	(0.134)	(0.066)
Received other agricultural training (=1)	0.146*	−0.138*	−0.008
	(0.086)	(0.084)	(0.055)
**Individual and household characteristics**			
Highest educational level, male respondent	0.100**	0.006	−0.107***
	(0.041)	(0.039)	(0.033)
Highest educational level, female respondent	−0.018	−0.012	0.030
	(0.057)	(0.053)	(0.038)
Married (=1), male respondent	−3.766	2.691	1.075
	(328.127)	(363.815)	(240.055)
Married (=1), female respondent	−0.565	−0.590	1.155
	(136.304)	(82.215)	(218.518)
Age (years), male respondent	0.007	−0.001	−0.006
	(0.006)	(0.005)	(0.004)
Age (years), female respondent	−0.005	−0.003	0.008*
	(0.006)	(0.006)	(0.004)
Severely Food Insecure (FIES)	−0.009	−0.007	0.016
	(0.063)	(0.060)	(0.038)
Household size	−0.003	0.004	−0.001
	(0.013)	(0.012)	(0.008)
*Asset/wealth quintile (reference = poorest)*			
Quintile 2	−0.014	−0.031	0.044
	(0.095)	(0.092)	(0.058)
Quintile 3	−0.057	−0.053	0.110*
	(0.096)	(0.091)	(0.060)
Quintile 4	−0.051	−0.029	0.080
	(0.094)	(0.088)	(0.062)
Quintile 5 (Richest)	0.027	−0.123	0.096
	(0.100)	(0.095)	(0.065)
**Observations**	357	357	357
**Pseudo R-squared**	0.116	0.116	0.116
*Households in which empowerment scores are equal (% of total)*	193 (53.91)		
*Households in which man is more empowered (% of total)*		122 (34.08)	
*Households in which woman is more empowered (% of total)*			43 (12.01%)

*Source*: Authors calculations

^a^Gender parity is defined as the woman being equally or more empowered than the main man in the household; estimated using logistic regression

^b^Estimated using multinomial logit, with base defined as households where woman and man are equally empowered. Marginal effects reported, standard errors in parentheses. (=1) represents dummy variables and coefficients denote the effect of a discrete change in the dummy variable from 0 to 1

* p < 0.10, ** p < 0.05, ***p < 0.01

See additional notes to Table 9

#### Bangladesh

Levels of individual empowerment differ by both gender and the node of the value chain (Table 3). Relative to the base category of agricultural producer households, women in both entrepreneur and wage-earner households have lower empowerment scores and are significantly less likely to be empowered. In contrast, men in both entrepreneur and wage-earner households are significantly more likely to be empowered and to have higher empowerment scores than men in producer households.

Though small in number, women in women-only households are more likely to be empowered and have slightly higher empowerment scores relative to women in dual-headed households. Women’s and men’s empowerment is positively correlated with educational attainment in our Bangladesh sample, but marital status, household size and receipt of cash or in-kind transfers are not significantly correlated with empowerment for either gender.

Women and men in wealth quintiles 2, 3 and 4 do not differ significantly from their counterparts in the poorest wealth quintile, either in the likelihood of being empowered or in the empowerment scores, with the exception of the empowerment score for men in wealth quintiles 3 and 4. However, women in the richest quintile are 6 percentage points less likely to be empowered than women in the poorest quintile (*p* < 0.01) and have an empowerment score than is 0.011 points lower (p < 0.01). In contrast, men in the richest quintile have, on average, an empowerment score that is 0.013 (p < 0.01) points higher than that of men in the poorest quintile. This decrease in women’s empowerment across the wealth gradient is consistent with other work in Bangladesh (Mahmud et al., 2012).

The specific node of the value chain the household is engaged in is strongly correlated with household wealth. Wage-earner households, typically without any land of their own, tend to come from the bottom two wealth quintiles. Agricultural producer households come predominantly from the middle three wealth quintiles, and entrepreneur households from the two richest, consistent with the idea that entrepreneurship is inherently risky. While it is difficult to disentangle the wealth effect from that of the specific node of the value chain, it does not appear that engaging in entrepreneurship—arguably a higher value node than production or wage labor—necessarily implies greater levels of women’s empowerment. If entrepreneurship is confined to small-scale, low-return activities, it may not bring about desired changes in women’s empowerment.

Compared to the base category of producer households, entrepreneur and wage earner households perform worse on intrahousehold measures (Table 4). Entrepreneur households are 21 percentage points (pp) less likely to achieve parity (*p* < 0.01). It is also 21 pp. less likely that the man and woman are equally empowered (p < 0.01), 21 pp. more likely that the man in the entrepreneur household is more empowered than the woman (p < 0.01), and 9 pp. less likely that the woman is more empowered than the man. The patterns for wage earner households are very similar, though the magnitudes are larger for every outcome measure. This corroborates our interpretation that producer households in the middle of the wealth spectrum display greater equity between men and women.

We find no correlation between intrahousehold measures of empowerment and men’s or women’s levels of education, household size or receipt of cash or in-kind transfers. There is some evidence that wealth is negatively correlated with intrahousehold empowerment measures. Compared to the poorest quintile, household in the richest quintile are 20 pp. less likely to achieve parity (*p* < 0.01) and 27 pp. more likely to have a man who is more empowered than the woman. Households in quintile 4 display similar trends though with smaller magnitudes; they are 9 pp. less likely to achieve parity (*p* < 0.05) and are 12 pp. (p < 0.05) more likely to have a man who is more empowered than the woman. Quintiles 2 and 3 are, for the most part, indistinguishable from the poorest quintile on intrahousehold measures of empowerment.

#### Philippines

Tables 5 and 6 present similar regressions for the Philippines, indicating again that empowerment differs across nodes and types of value chains. Both women and men are least empowered in the coconut value chain, and most empowered in the seaweed value chain. Women who are engaged in processing have lower empowerment scores compared to those engaged in production and trading. The low scores of women processors may arise from their engagement in low-value and time-demanding processing activities in coconut and abaca and the poor work conditions in abattoirs. In terms of market participation, men who participate in wage employment are 8 pp. less likely to be empowered than those who are not (*p* < 0.05); having one’s own business may involve greater autonomy than wage work.

Being a woman in a woman-only household is not significantly correlated with greater empowerment. Women’s and men’s empowerment is positively correlated with their own education, age, being married, access to extension services, and access to community programs and projects, with some differences by gender. Education and extension services are more strongly associated with men’s empowerment than women’s. Although earlier studies in similar contexts (e.g., Samarakoon & Parinduri, 2015 for Indonesia) point to the positive association between education and women’s empowerment, in our study setting, the weaker association between education and women’s empowerment is likely attributable to the higher proportion of women who have completed secondary schooling or higher compared to men, not unusual in the Philippines. Similarly, access to extension services is associated with a 12 pp. increase in likelihood of the man being empowered and a 7% increase in their empowerment score; these effects are smaller for women with access to extension, with access to extension associated with only a 5 pp. increase in the woman’s likelihood of being empowered, and a 4% increase in her empowerment score. Even if education increases women’s bargaining power within their households, it may be insufficient to change deeply rooted societal attitudes. Interestingly, there is no strong relationship with household wealth; a woman is more likely to be empowered if she belongs to the top wealth quintile, but none of the other wealth categories are significant.

Table 6 presents regressions on the correlates of intrahousehold inequality measures in the Philippine sample. The node of the value chain matters: men’s participation in trading is correlated with a higher likelihood of his being more empowered, whereas the woman’s participation in trading is correlated with a lower likelihood that the man is more empowered. Participation in trading may involve more direct access to sales proceeds on higher value products, as well as more engagement with other market actors, which may itself be empowering. Among the four value chains, participation in the abaca value chain is correlated with a higher likelihood of the man being more empowered, relative to a condition of gender equality. The male respondent’s participation in nonfarm activities and wage employment (relative to agricultural production) is correlated with a lower likelihood that he is more empowered, relative to a condition of gender equality. Nonfarm work and wage employment may be relatively low-return sectors for men in these contexts.

Households are more likely to achieve gender parity if the woman is more educated and is older, though the marginal effects are relatively small. Participation in and access to extension and community programs often show opposite signs by gender, suggesting that increasing men’s and women’s access to services may offset each other. Men’s access to extension services increases the likelihood that the man is more empowered by 28 pp. (*p* < 0.10) (and lowers the likelihood that the woman is more empowered by 52 pp. (*p* < 0.001), and therefore is correlated with greater likelihood of inequality. Women’s access to extension services is associated with a 32 pp. (p < 0.10) reduced likelihood that the man is more empowered, and a 5.2 pp. (*p* < 0.05) increase in the likelihood that men and women are equally empowered. Surprisingly, women’s own access to community programs is associated with a 40 pp. (p < 0.10) increased likelihood that the man is more empowered, and a 6.3 pp. decrease in the likelihood of achieving gender parity. Men’s access to community programs is correlated with a 32 pp. decrease in the likelihood that the woman is more empowered, but this is only weakly significant. If extension services and community programs are targeted to specific individuals within the household, this could worsen gender inequality and disempower their partners, unintentionally limiting households’ participation in these programs compared to programs that could potentially empower both men and women.

#### Benin

Table 7 presents regressions on women’s and men’s individual empowerment scores in the Benin sample, as a function of individual and household characteristics. The regressions for Benin include additional indicators of access to agricultural training and severe food insecurity according to the Food Insecurity Experience Scale (FIES).

There were no significant differences among the empowerment scores of women who participated in soy and poultry processing compared to those participating in rice processing. The same finding was true for the men who lived in their households. Women participating in composting had a higher likelihood of being empowered relative to those participating in rice processing, and the men living in households with women who participated in compost experienced an increased likelihood of being empowered. The positive correlations are evident for women’s and men’s empowerment scores as well. Receiving ATVET4W training is correlated with a higher likelihood of being empowered and higher empowerment scores, but only for men. Because the sample does not include a randomly selected control group, this coefficient does not indicate program treatment effects.

Women residing in women-only adult households, older women, and women with higher educational attainment are significantly more likely to be empowered. There is no correlation between men’s educational attainment and their own empowerment. Belonging to a larger household is weakly correlated with a higher empowerment score for men. Greater household wealth is correlated with empowerment for men; it is only in the top two quintiles that we observe positive correlations between wealth and women’s empowerment (both the likelihood of being empowered and the empowerment score).

Very few covariates show significant associations with the household achieving gender parity (Table 8). In households where women process soy, there is a higher likelihood of achieving gender parity, while being involved in composting is weakly correlated with the woman being less empowered. Receiving ATVET4W training has a weak positive correlation with the woman being more empowered.

Interestingly, while a higher educational level of the male respondent is associated with a higher probability of a woman’s being empowered, a higher educational level of the woman is associated with a lower probability that the man is more empowered than her and a higher likelihood that the household achieves gender parity. Overall, the correlations of parity measures with wealth are weak.

#### Malawi

In Malawi, the main value chain the household is involved in does not appear to be significantly correlated with individual men’s or women’s likelihood of being empowered or their empowerment scores (Table 9), which may be attributable to the types of value chains targeted in the study or the types of households selected to participate in an intervention that targeted couples. The ATVET4W training occurred a relatively short period of time before the survey in Malawi; perhaps because of this, receiving the ATVET4W training does not seem to be significantly associated with women’s empowerment in this early phase of implementation, although having received other agricultural training is associated with greater empowerment for both men and women.

Belonging to a woman-only household is not significantly associated with a higher likelihood of empowerment for women but is weakly associated with higher empowerment scores for women. Higher educational levels are empowering for both women and men alike, but there does not appear to be a wealth gradient with respect to empowerment for either men or women. A woman’s own age is correlated with a higher empowerment score, but not a greater likelihood of her being empowered.

Finally, we examine correlates of intrahousehold inequality in Table 10. Once again, the main value chain for the household is not significantly correlated with gender parity, or with the man or woman being more empowered. In contrast to the results in Table 9, ATVET4W training is strongly positively correlated with the woman being more empowered than the man and negatively correlated with the man being more empowered than the woman, which could be a consequence of the types of households that were selected for this training. Provision of other agricultural training is weakly correlated with the household achieving gender parity, but also with the man being less empowered, which could be the result of previous training programs targeting women but not men.

While higher educational levels for men are correlated with gender parity, they are also associated with a lower likelihood that the woman is more empowered; women’s education has no significant correlation with the gender parity measures. Women’s age is also an important factor that is correlated with gender parity; the woman is more likely to be empowered than the man in households where the female respondent is older. In contrast, a household where the man is older is likely to be one where the woman is less empowered than the man. There is also a positive correlation between wealth and the woman being more empowered, but only in quintiles 3 and 5.

### Summary of findings from the four countries

Our analysis across the four countries suggest that entrepreneurship is not necessarily empowering for rural women. In our Bangladesh sample, for example, men in entrepreneurial households are more likely to be empowered, but women in those households are not. This may relate to gender norms in Bangladesh as well as the scale of the enterprise in which women entrepreneurs are involved. Small-scale enterprises with low returns (like trading) may not be empowering. Greater involvement in the market is also not necessarily associated with gender equality. For example, in our Benin sample, a decrease in the amount of the household’s main commodity sold was correlated with higher gender equality. Some commodities may provide more opportunities for empowerment. For example, high return export sectors (seaweed in the Philippines) or those that do not require large-scale operations or that can be grown close to the home (swine in the Philippines), could reduce tradeoffs between market and caregiving work.

In our samples, training and extension services are usually associated with greater empowerment but may differentially benefit men and women. In the Philippines, access to extension services seems to have a stronger correlation with men’s than women’s empowerment. In Benin, receiving ATVET4W training was associated with a higher likelihood of only the man being empowered. In Malawi, receiving the ATVET4W training was not significantly associated with individual men’s and women’s empowerment, but was associated with a higher likelihood that the woman is more empowered and that the man is less empowered, which may be a consequence of the proximity of the training to the survey and the types of couples selected for the program. In the Malawi sample, receiving other types of agricultural training was positively correlated with the probability of being empowered, with higher empowerment scores for both men and women, and with greater gender parity.

Education is associated with greater empowerment of both men and women, but the “empowerment returns” to education vary across contexts. In Bangladesh, for example, both men’s and women’s education levels are associated with a higher empowerment score, but this association is only weakly significant for women and highly significant for men. In the Philippines, education is more strongly associated with men’s empowerment than women’s empowerment. In Benin and Malawi, a woman’s own education levels are positively associated with women’s empowerment scores, but men’s education levels are not associated with their empowerment scores in Benin. With the exception of the Philippines, where women’s education is positively associated with achieving gender parity, men’s and women’s schooling is not significantly associated with the likelihood that a woman is as empowered or more empowered than the man in her household.

We also find that greater wealth is not always correlated with empowerment for women. Women’s empowerment is inversely correlated to wealth in our Bangladesh sample—but positively so in the Philippines and Benin samples. This finding implies that we cannot assume that women are going to become more empowered from wealth alone. In our Bangladesh sample, being in the top two wealth quintiles is associated both with a lower likelihood that the household attains gender parity and a higher likelihood that the man is more empowered. In contrast, there is no wealth gradient with respect to intrahousehold inequality measures in the other three countries in our sample.

All in all, culture and context determine whether participation in value chains—and which node of the value chain—is empowering. This suggests that food systems and value chains interventions that seek to empower women should consider the social and cultural contexts in which these food systems operate, so that interventions “do no harm” and do not exacerbate existing gender inequalities.

## Concluding remarks

This paper aimed to examine the factors conducive to women’s empowerment and gender equality within key agricultural value chains. We draw on Njuki et al.’s (2021) Gendered Food Systems framework that describes food systems as comprised of three components—value chains, the food environment, and consumer behavior—each of which interacts with and is affected by women’s empowerment and gender equality. Njuki et al.’s (2021) review finds that the interaction between value chains and women’s empowerment is the least studied of the three components. Our paper makes two contributions toward closing this knowledge gap. First, it uses a validated metric of women and men’s empowerment that was specifically adapted for value chain research and permits the calculation of both absolute and relative levels of empowerment within a household. Second, it applies this metric to data from four countries and multiple commodity value chain contexts and from male and female actors engaged at different nodes of these value chains. Doing so permits us to generate a rich set of contextual insights into the factors correlated with greater women’s empowerment and gender equality.

### Key findings

Using data from four countries—Bangladesh and the Philippines in Asia, and Benin and Malawi in Africa —we provide insight into correlates of aggregate empowerment measures. As expected, findings vary by context. There are considerable differences across countries in aggregate empowerment and gender equality within the household; there are also sizeable differences across value chains and nodes of those value chains *within* a country. Entrepreneurship—often engaged in by wealthier households with greater ability to take risks—is not necessarily empowering for women; nor is greater involvement in the market necessarily correlated with increased gender equality.

Household and individual characteristics also matter. Greater household wealth, as proxied by asset ownership, is not necessarily empowering for women. In fact, we find an inverse correlation of wealth with women’s empowerment in Bangladesh, debunking the assumption that women in wealthier households are more empowered. Education is positively correlated with higher empowerment of both men and women, but the strength of this association varies—strong in the Philippines and Malawi, and weaker in Bangladesh and Benin. Surprisingly, the gap between men’s and women’s empowerment scores is less sensitive to education than the absolute empowerment scores for both sexes. Training and extension services are generally positively associated with empowerment but could also exacerbate the inequality in empowerment between men and women in the same household.

### Limitations and areas for future research

Our conclusions are based on empirical analyses of household datasets from a small set of countries that were selected with the goal of comparing different commodities or nodes, or for program evaluation. We do not track households over time, and one limitation of our observational analysis is our inability to attribute causation. All relationships estimated should be interpreted as associations or correlations.

Another limitation is our focus on a limited set of countries in Asia and Africa. The Latin America and the Caribbean region is an obvious exclusion, despite the high degree of women’s involvement in agriculture and integration into markets. Further work in this geographic area is needed.

A third limitation is the use of the pro-WEAI+MI, a metric developed to measure women’s and men’s absolute and relative empowerment along value chains. Food systems encompass more than value chains, specifically the food environment and consumer behavior and how they interact with a variety of individual and structural factors, all of which are relevant to women’s empowerment and gender equality. A more comprehensive measure would capture other drivers of empowerment in food systems that may operate at a higher scale than the individual or the household.

Our analysis is also limited to small-scale producers and entrepreneurs, owing to the sampling design of our value chain studies. Gender dynamics may change as these small-scale food systems actors become more integrated into the market and increase the scale of their enterprises. In some markets, such as export markets for key agricultural commodities and markets for organically grown agricultural products, private sector initiatives, such as Voluntary Sustainability Standards (VSS), have arisen to promote economically, environmentally, and socially sustainable production and trade practices. These VSS can be leveraged to promote gender equality; Sexsmith (2019) provides helpful guidelines that are applicable to a broad range of food systems actors. These guidelines, which are consistent with our findings, cover household food security, women’s rights to agricultural productive resources, gender equality in education, women’s unpaid domestic labor, women’s decision-making and empowerment, and decent work. Investigating whether and how the application of these guidelines across a range of private sector actors affects gender dynamics and women’s empowerment within the participating households and producer and marketing organizations is a promising area for future work.

Finally, we are unable to make conclusions about the impact of gender-sensitive interventions because only two of our cases were drawn from impact evaluations, and we did not attempt to analyze the impact of these interventions in this paper. However, it seems reasonable to hypothesize that gender-sensitive interventions, which account for the interaction of gender with other aspects of the food system, and gender transformative interventions, which make the effort to transform and change existing gender norms and barriers, may be more successful than gender accommodative interventions, which simply target women but do not engage with the system as a whole. Tracking the performance of food systems interventions that explicitly incorporate gender—whether through gender-transformative, sensitive, or accommodative approaches—will provide crucial information on promising approaches that empower women and reduce gender inequality.

## Appendix 1. Pro-WEAI indicators

**Table:**

**Indicator**	**Definition of adequacy in pro-WEAI**
	*Intrinsic Agency*
Autonomy in income	More motivated by own values than by coercion or fear of others’ disapproval: *Relative Autonomy Index*^1^ score > =1
Self-efficacy	“Agree” or greater on average with self-efficacy questions: *New General Self-Efficacy Scale*^*C*^ score > =32
Attitudes about intimate partner violence against women	Believes husband is NOT justified in hitting or beating his wife in all 5 scenarios:^2^ 1).She goes out without telling him 2).She neglects the children 3).She argues with him 4).She refuses to have sex with him 5).She burns the food
Respect among household members	Meets ALL of the following conditions related to another household member: 1).Respondent respects relation (MOST of the time) AND 2).Relation respects respondent (MOST of the time) AND 3).Respondent trusts relation (MOST of the time) AND 4).Respondent is comfortable disagreeing with relation (MOST of the time)
	*Instrumental Agency*
Input in productive decisions	Meets at least ONE of the following conditions for ALL of the agricultural activities they participate in, whether related to production, processing, and marketing activities. 1).Makes related decision solely, 2).Makes the decision jointly and has at least some input into the decisions 3).Feels could make decision if wanted to (to at least a MEDIUM extent)
Ownership of land and other assets	Owns, either solely or jointly, at least ONE of the following: 1).At least THREE small assets (poultry, nonmechanized equipment, or small consumer durables) 2).At least TWO large assets 3).Land
Access to and decisions on financial services	Meets at least ONE of the following conditions: 1).Belongs to a household that used a source of credit in the past year AND participated in at least ONE sole or joint decision about it 2).Belongs to a household that did not use credit in the past year but could have if wanted to from at least ONE source 3).Has access, solely or jointly, to a financial account
Control over use of income	Has input in decisions related to how to use BOTH income and output from ALL of the agricultural activities they participate in AND has input in decisions related to income from ALL non-agricultural activities they participate in, unless no decision was made
Work balance	Works less than 10.5 h per day: Workload = time spent in primary activity + (1/2) time spent in childcare as a secondary activity
Visiting important locations	Meets at least ONE of the following conditions: 1).Visits at least TWO locations at least ONCE PER WEEK of [city, market, family/relative], or 2).Visits least ONE location at least ONCE PER MONTH of [health facility, public meeting]
*Collective Agency*	
Group membership	Active member of at least ONE group
Membership in influential groups	Active member of at least ONE group that can influence the community to at least a MEDIUM extent

^1^The Relative Autonomy Index (RAI), based on self-determination theory, is a measure of internal and external motivations that determine person’s decisions

^2^The New General Self-efficacy Scale (NGSE) is a validated scale to measure self-efficacy, or a person’s capabilities and ability to reach their goals

Note: Table source is Malapit, Hazel, Agnes Quisumbing, Ruth Meinzen-Dick, Greg Seymour, Elena M Martinez, Jessica Heckert, Deborah Rubin, Ana Vaz, Kathryn M Yount, and Gender Agriculture Assets Project Phase. 2019. “Development of the Project-Level Women’s Empowerment in Agriculture Index (pro-WEAI).” World Development 122: 675–92
